# New Trends to Treat Muscular Atrophy: A Systematic Review of Epicatechin

**DOI:** 10.3390/nu16020326

**Published:** 2024-01-22

**Authors:** Iris Jasmin Santos German, Karina Torres Pomini, Jesus Carlos Andreo, João Vitor Tadashi Cosin Shindo, Marcela Vialogo Marques de Castro, Claudia Rucco P. Detregiachi, Adriano Cressoni Araújo, Elen Landgraf Guiguer, Lucas Fornari Laurindo, Patrícia Cincotto dos Santos Bueno, Maricelma da Silva Soares de Souza, Marcia Gabaldi, Sandra Maria Barbalho, André Luis Shinohara

**Affiliations:** 1 Department of Biological Sciences (Anatomy), School of Dentistry of Bauru, University of São Paulo, (FOB-USP), Alameda Doutor Octávio Pinheiro Brisolla, 9-75, Bauru 17012-901, São Paulo, Braziljvshindo@usp.br (J.V.T.C.S.);; 2 Postgraduate Program in Structural and Functional Interactions in Rehabilitation, University of Marilia (UNIMAR), Marília 17525-902, São Paulo, Brazil; karinatorrespomini@gmail.com (K.T.P.); marcelavialogo@hotmail.com (M.V.M.d.C.); adrianocressoniaraujo@yahoo.com.br (A.C.A.); elguiguer@gmail.com (E.L.G.); smbarbalho@gmail.com (S.M.B.); 3 Department of Biochemistry and Pharmacology, School of Medicine, University of Marília (UNIMAR), Avenida Hygino Muzzy Filho, 1001, Marília 17525-902, São Paulo, Brazil; lucasffffor@gmail.com (L.F.L.); maricelma.soares.souza@gmail.com (M.d.S.S.d.S.);; 4 Department of Animal Sciences, School of Veterinary Medicine, University of Marília (UNIMAR), Avenida Hygino Muzzy Filho, 1001, Marília 17525-902, São Paulo, Brazil

**Keywords:** epicatechin, skeletal muscle, muscular atrophy, catechins, myogenic regulatory factors

## Abstract

Epicatechin is a polyphenol compound that promotes skeletal muscle differentiation and counteracts the pathways that participate in the degradation of proteins. Several studies present contradictory results of treatment protocols and therapeutic effects. Therefore, the objective of this systematic review was to investigate the current literature showing the molecular mechanism and clinical protocol of epicatechin in muscle atrophy in humans, animals, and myoblast cell-line. The search was conducted in Embase, PubMed/MEDLINE, Cochrane Library, and Web of Science. The qualitative analysis demonstrated that there is a commonness of epicatechin inhibitory action in myostatin expression and atrogenes MAFbx, FOXO, and MuRF1. Epicatechin showed positive effects on follistatin and on the stimulation of factors related to the myogenic actions (MyoD, Myf5, and myogenin). Furthermore, the literature also showed that epicatechin can interfere with mitochondrias’ biosynthesis in muscle fibers, stimulation of the signaling pathways of AKT/mTOR protein production, and amelioration of skeletal musculature performance, particularly when combined with physical exercise. Epicatechin can, for these reasons, exhibit clinical applicability due to the beneficial results under conditions that negatively affect the skeletal musculature. However, there is no protocol standardization or enough clinical evidence to draw more specific conclusions on its therapeutic implementation.

## 1. Introduction

There has been a growing interest in the study of catechins and the properties reported in the scientific literature related to their antioxidant, regenerative, and anti-inflammatory capacity [1,2,3].

There are four major subclasses of catechins: Epicatechin (EC), Epigallocatechin (EGC), Epicatechin gallate (ECG), and Epigallocatechin gallate (EGCG) [2]. Among the different catechins, EC and EGCG have greater effects on the skeletal musculature. Catechins exhibit beneficial effects on skeletal muscle, specifically on myoblast differentiation, but only epicatechin promotes mitochondrial biogenesis and angiogenesis, as described by Li et al. [4].

**Figure 1:**
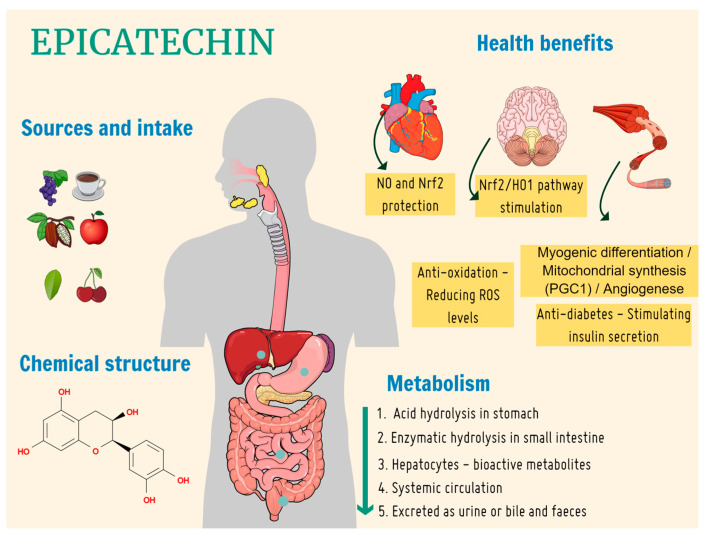
Main food and beverage sources, chemical structure, metabolic route, and biological properties of epicatechin. NO, nitric oxide; Nrf2, nuclear factor-like 1; HO, heme oxygenase; PGC1α, peroxisome proliferator-activated receptor γ coactivator 1-alpha; ROS, reactive oxygen species.

EC is a polyphenolic compound found at high concentrations in certain fruits and vegetables, including tea leaves, black grapes, chocolate, apples, raspberries, and cherries [2,5,6]. EC is mainly extracted from green tea (*Camellia sinensis*) [4,5]. The consumption of such polyphenols has been linked to several positive effects on diseases involving oxidative stress, such as cancer, diabetes, cardiovascular, and degenerative diseases [7,8]. Figure 1 shows the biological properties of epicatechin.

More recently, EC has shown the potential capacity to mitigate and delay the loss of muscle mass in diseases that affect the musculoskeletal system [4,9,10,11].

The role of dietary supplements in activating specific pathways that mitigate or neutralize muscle atrophy in patients with diseases that participate in the same muscle atrophy signal pathway has been highlighted [12]. For example, sarcopenia is a skeletal disorder that negatively affects muscle mass and is closely related to cardiovascular disease and other chronic diseases [13].

Muscular atrophy is a musculoskeletal disease characterized by the loss of cell size. In this process, there is a decrease in proteins and organelles and an increase in the expression of genes associated with muscle atrophy, accelerating protein catabolism and compromising function and performance [4]. In addition, muscle atrophy occurs in response to different conditions such as diabetes, cardiac failure, cancer, fasting, aging [14,15], obesity, rheumatoid arthritis, and physical inactivity [13].

The regulation of skeletal muscle growth occurs via the IGF-PI3K-AKT pathway. The enzyme phosphatidylinositol-3-kinase (PI3K) allows glycogen to enter cells by facilitated diffusion; subsequently, the pyruvate dehydrogenase kinase (PDK1) transmits the PI3K signal, acting as a second messenger. Then, AKT (Protein kinase B) acts on protein signaling pathways, activating the largest signal integrative pathway, mTOR, which, through the substrate S6K1 and the binding protein 4EBP1, regulates protein quality, promoting muscle growth [4].

During protein degradation, the Ubiquitin Proteasome System is expressed, activating autophagic genes via forkhead box, subgroup O (FOxO), and triggering genes associated with muscular atrophy, atrogin-1 (MAFbx), muscle RING-finger protein-1 (MURF1), and LC3 (Light Chain) [4,10].

In this muscle restructuring, several myogenic regulation factors participate, mainly in the proliferation stages. Myoblast determination protein (MyoD) and Myogenic factor 5 (Myf5) are involved, which are necessary for the determination of the myobrast; later, in the differentiation phase, myogenin and Miogenic regulatory factor 4 (MRF4) are expressed [9].

Oral nutrition supplements benefit patients with malnutrition associated with chronic diseases and hospitalized patients during recovery [12,16,17]. Vitamin supplementation, especially vitamin D, is essential in muscle strength and performance [18,19,20], and in regulating bone mineral density (BMD) [21,22].

As described by Savary-Auzeloux et al. (2013) [23], nutritional supplementation of antioxidants/polyphenols contributes to the signaling of different factors involved in protein synthesis, even in absence of physical exercise and/or skeletal muscle recovery from disuse.

Furthermore, dietary supplements activate and modulate depending on their specific composition and standard mechanism of action. Some common biochemical pathways involved include:

- mTOR signaling: many dietary supplements target the mammalian rapamycin (mTOR) signaling pathway, related to protein turnover and autophagy (a process of recycling resulting in degradation of the body’s own tissue) [24];
- AMP-activated protein kinase (AMPK) pathway: AMPK is a metabolic pathway that regulates energy metabolism and cellular energy homeostasis. Some supplements can activate the AMPK pathway to promote processes using fat as an energy source [25];
- nuclear transcription factor kappa B (NF-kB) pathway: NF-kB is a pathway in inflammatory and immune response. Supplements can modulate the NF-kB pathway to regulate inflammation and promote a healthy immune response [26];
- peroxisome proliferator-activated receptors (PPAR) pathway: PPARs are a family of receptors that regulate metabolism of lipids and energy homeostasis. Oral nutrition supplements can activate PPARs to modulate metabolism and the inflammatory response [27].

Clinical studies have related the catechins to the protective effects of the skeletal musculature by inducing myogenic differentiation and improving muscle structure and function [28,29].

In the skeletal muscle, EC acts directly and indirectly in the protein synthesis signaling [30], reduces the catabolic effect [31,32] by stimulating the PI3K/Akt pathway and by inactivating the autophagic genes FoxO, MAFbx, and MuRF [2]. This mechanism of action of EC in the muscle occurs by inhibiting the degradation proteins and increasing mitochondrial biogenesis [6,33].

Research in animals that received catechins presented an increase in the Muscle Regulatory Factors (MRF), including MyoD, Myf5, and Myogenin, and a decrease occurred in myostatin, a protein identified as modulatory of the primary catabolic pathways, participating in the signaling that regulates the muscular atrophy [9,34].

Due to the great benefits of EC supplementation and its clinical relevance in the treatment of diseases that affect the skeletal muscles, it is crucial to summarize the evidence available on the effects of this polyphenol using the search strategy. Despite the positive effects of EC, there are conflicting results and non-standardized therapeutic protocols.

Our systematic review identifies substantive gaps in the current understanding of the effects of epicatechin on muscle atrophy, providing a solid foundation to drive future investigations. By highlighting inconsistencies and deficiencies in the existing literature, our approach not only consolidates existing knowledge but also catalyzes the pressing need for subsequent research to fill these gaps and enhance our understanding of the clinical applicability of EC in skeletal muscular atrophy.

In this context, this systematic review aimed to analyze the existing literature, addressing epicatechin supplementation’s molecular effects and clinical protocol to counteract muscle atrophy in humans, animals, and myoblast cell lines.

## 2. Materials and Methods

### 2.1. Data Extraction Methods

This systematic review was substantiated through the PICO strategy [35]—P: use of EC in humans and animals; I: application of EC in muscular atrophy; C: comparison with the control/placebo group; O: effects on the skeletal musculature. The choice of the PubMed/MEDLINE, Web of Science, Embase, and Cochrane Library databases were based on the PICO strategy in order to evaluate the clinical protocols and protein turnover effects of EC supplementation on skeletal musculature atrophy condition.

All databases were searched in August–September 2023, including the terms registered in the Medical Subject Headings (MeSH): Catechin, muscular atrophy, muscle regeneration, epicatechin, muscle, and damage by associating the following keywords with no restrictions concerning the year of publication of the articles: “Catechin and muscular atrophy”, “Epicatechin and muscle regeneration”, “Epicatechin and muscle and damage”.

Two authors conducted the search to achieve a more reliable selection of articles in the databases.

Two independent authors carried out the selection of articles and the evaluation of the full text. No filters were used in the databases in order to avoid losing relevant studies. In addition, studies from gray literature were analyzed to identify potentially relevant studies for this systematic review. Furthermore, experts in the field were contacted to obtain pertinent information or recommendations for important articles.

The articles were selected considering the eligibility criteria and PRISMA checklist [36].

### 2.2. Inclusion Criteria

- In vivo and in vitro studies that evaluated EC in the treatment of muscular atrophy.
- Studies with specifications of the dosage of EC used, treatment time, and administration route.
- Systematic literature reviews.

### 2.3. Exclusion Criteria

- Articles that used another type of catechin or flavonoid.
- Duplicated articles.
- Studies that did not analyze EC effects on skeletal musculature.

**Figure 2:**
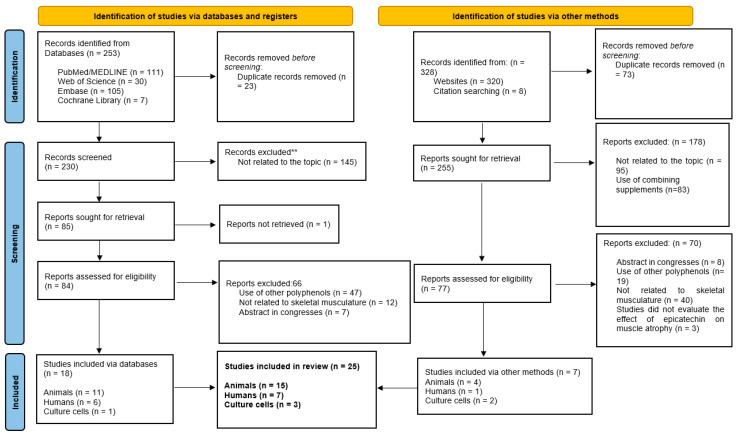
PRISMA 2020 flow diagram. Search design strategy in the databases and other sources. **: records identified in the databases but not related to this review topic.

To search, the keywords were combined in each database. The articles were selected by title and then by reading the abstracts; thus, they were organized, and subsequently, the articles were restricted according to the eligibility criteria, following the proposed methodology and the PRISMA checklist [36]. Figure 2 shows the search design strategy in the databases.

## 3. Results

### 3.1. Data Synthesis Methods-Search Results

A total of 253 articles were identified in search for this systematic review. In PUBMED/MEDLINE database, 111 articles were verified, 30 studies in Web of Science, 105 in Embase, and 7 articles in Cochrane Library. After removing the duplicated articles, 230 remained, of which 145 studies were eliminated as they were unrelated to the subject of investigation. Of the 85 articles remained, 1 record could not be located, and no response was received when the authors were contacted. As such, 84 reports were assessed for eligibility and, after reading, 66 articles were excluded: 47 used another type of polyphenol, 12 studies used EC in other muscle tissue, and 7 abstracts were from congresses. Thus, 18 articles were included (11 studies in animals, 6 in humans, and 1 in vitro research).

In addition, 328 reports were located on websites and citation trackers. After removing duplicate records, 255 articles were screened; 178 were excluded and the remaining 77 reports were advanced to reading the full text. Of these, 70 articles were excluded. As such, only seven reports matched the eligibility criteria.

Finally, 25 articles were included in this systematic study (15 studies in animals, 7 in humans, and 3 in vitro articles). Table 1, Table 2 and Table 3 show the main information about the 25 studies selected in humans, animals, and in vitro, respectively.

### 3.2. Risk of Bias in the Studies

#### Risk of Bias Assessment Methods

A reduced sample size was identified [37,42,43]; brief time assessment [40]; the data of the participant’s diet were not collected [41,42]; absence of an evaluation of EC consumption in different periods [37,38,39,40,41,42,45,46,48,49,50,51,54,55,57,58,59,60,61]; only one dose was studied [37,38,39,41,42,43,44,45,46,47,48,49,50,51,52,53,54,55,56,58,60,61]; absence of EC plasma concentrations [39,40,42,43,44,45,47,48,49,50,51,52,53,54,55,56,57,58]; difference of the period of euthanasia in the groups [52]; animal model for longevity studies–db/db BKS.Cg- mice, a mutation of the C57BLKS/J lineage [44]; the effect of EC interruption was not evaluated [37,38,39,40,41,42,43,44,45,46,48,49,50,51,52,53,54,55,56,57,58,59,60,61]; the participants’ gender was not reported [48]; No control group [43]. The previous data are presented in Table 4.

## 4. Discussion

Coadjuvant approaches in musculoskeletal diseases have provided beneficial results to the quality of life of the affected individuals [62,63,64].

Epicatechin supplementation has exhibited promising clinical applicability in the regeneration of muscle tissue [6,23,28,29,33,34,37,38,39,41,42,43,44,45,46,47,48,49,50,51,52,53,54,55,56,57,58,59,60,61]. Thus, this systematic review intended to evaluate the effects of EC as dietary supplementation on skeletal muscle atrophy.

This systematic review showed the effect of epicatechin on increasing follistatin [37,39,42,43,48,54] and decreasing myostatin [9,34,37,39,42,48,57], activating mitochondrial biogenesis [42,47,49,59], and muscle capillary [45,46,47,49] due to VEGF stimulation [45,46,47,49]. In skeletal muscle, epicatechin suppresses the expression of atrogenes induced by FoxO [2,49,51,53,54] and improves muscle performance when combined with physical activity [52,54]. In addition, the major protein synthesis pathway (AKT/mTORC1) [2,30,31,32,51,55] and specific tissue markers that control myogenic differentiation, such as Myf5 [9,34,37,42,48], MyoD [48,56,58], and myogenin [48,56,57,58,60], were stimulated by epicatechin.

The main pathways associated with muscle atrophy involve IGF1-Akt-FoxO signaling. This pathway also participates in ROS overproduction and calcium metabolism [65]. Additionally, IGF is present in different tissues of the human body [66]. IGF is essential in the regenerative capacity and muscle growth by AKT phosphorylation that controls mTOR pathway [66]. During the process that controls the size of the muscle fiber, genes that participate in muscle atrophy are activated, led by the main transcription factor, FoxO, and there is an increase in autophagic genes atrogin-1 (MAFbx) and MuRF1, therefore promoting protein degradation [66,67,68,69].

In addition, two proteolytic systems involved in the pathophysiology of muscle atrophy and regulate protein turnover and muscle homeostasis have been described: the ubiquitin proteasome system (UPS) and the autophagy system [69]. The main genes of the UPS system are MuRF1 and MAFbx (atrogin-1) [69]. An impairment of these systems can lead to an excessive activity of protein degradation and consequently compromise the contraction of myofibers [66].

In proteolytic activity, the calpain system participates in protein turnover during physical activities or disused muscles, regulating Ca^2+^ signaling and contributing to the USP system by assisting the degradation of sarcomeres’ proteins [65].

Another signaling pathway that acts as a negative regulator of muscle growth is the myostatin-Smad2/3 pathway [4]. Myostatin inhibits IGF1-AKT-mTOR signaling and synergizes the FoxO signaling pathway, leading to muscle atrophy [70,71]. The mechanism to prevent atrophic gene activation consists of increasing follistatin (antagonist protein of myostatin) to interrupt myostatin binding to the receptor [70,71,72,73].

**Figure 3:**
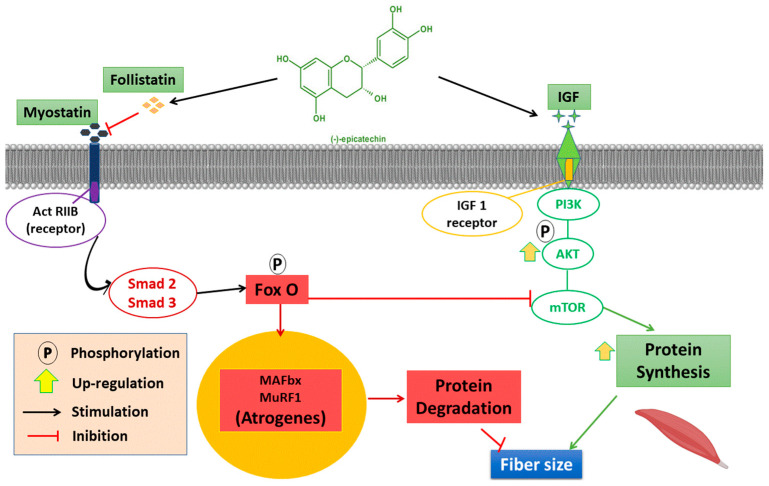
An overview of epicatechin effects on skeletal muscle. Modified diagram from Li et al. [4]. Act RIIB, myostatin receptor; Smad 2/3, mothers against decapentaplegic homolog 2; FoxO, forkhead transcription factor family; MAFbx, muscle atrophy F-box; MuRF1, muscle RING-finger protein-1; IGF, insulin-like growth factor-1; PI3K, phosphatidylinositol 3 kinase 9; AKT, Protein kinase B; mTOR, The mammalian target of rapamycin.

Seven articles in this review verified an increase in the follistatin/myostatin ratio [37,39,42,43,48,54,57]. Taub et al. [37] and Mafi et al. [39] noted an increment in the follistatin levels but no significant differences in myostatin. Besides, Schwarz et al. [38] did not observe any changes in myostatin expression and no benefits in the adaptations of anaerobic exercise. In addition, myostatin inexpression may be caused by the EC stimulus on the high plasma levels of testosterone in the skeletal muscle, causing myostatin suppression [74,75]. Figure 3 shows the general effects of epicatechin on protein synthesis and degradation.

As described by Li et al. [4] and Gutierrez-Salmean et al. [48], EC binds to the myostatin receptor, the C-terminus, interfering the expression and activity of myostatin.

Moreover, mitochondrial biogenesis is regulated by the expression of transcriptional coactivators, such as those from the PGC-1 family, in addition to the activity of AMPK, p38 MAPK, and TFAM signaling [4,25].

In this review, Lee et al. [49] and Gonzalez-Ruiz et al. [53] reported the effect of EC on the ubiquitin-proteasome system (UPS) through inactivation of the autophagic genes FoxO, MAFbx, and MuRF1, resulting in the blockage of the catabolic pathways in the skeletal muscle. However, some studies did not note significant effects on the reduction of protein MAFbx [54,55], the increase of follistatin, Pax7, and the decrease of atrophy markers myostatin [41], MURF and Fbox40 [55].

Epicatechin showed positive effects on the myogenic differentiation processes of tissue markers Myf5, MyoD, and myogenin [37,42,48,56,57,58,60]; in addition, EC increased the activation of AKT/mTORC1 signaling [51,55] and stimulated the myocyte enhancer factor 2A (MEF2A) expression [37,42,48,54].

Concerning cell proliferation and differentiation, epicatechin activated mitochondrial biosynthesis in the muscle fibers at the dosage of 100 mg per day for 3 months (in humans) [42]. In animals, PGC-1α was increased by EC at 2 mg/kg for 30 days [47], 2 mg/kg for 8 weeks [49], 2 mg/kg for 14 consecutive days [51], and 1000 mg/kg [57].

According to the existing studies, EC showed an action on the mitochondrial biogenesis of the skeletal muscle [4,6,28,76]. Such a mitochondrial induction mechanism, stimulated by EC, has been proposed by Moreno-Ulloa et al. [59] and seems to be caused by epicatechin bonding to receptor GPER (G protein-coupled estrogen receptor 1), expressed in several tissues of the human body, including in the metabolic homeostasis of the skeletal muscle [77].

In myoblasts cell-line, EC activated regulators of mitochondrial functions, such as the Succinate Dehydrogenase (COX-I/SDH-A) [59], the Peroxisome proliferator-activated receptor coactivator-1 (PGC-1), Acetyl-CoA carboxylase (ACC), and Mitochondrial transcription factor A (TFAM) [61]. In addition, EC increased the diameter of C2C12 myotubes [59,61].

Another factor involved in skeletal muscle metabolism is the density of the blood vessels, which has the function of supplying oxygen and metabolites through the capillaries [78]. Among the articles analyzed in this review, Hüttemann et al. [45], Ramirez-Sanchez et al. [46], Hüttemann et al. [47], and Lee et al. [49] noted a significantly higher increase of the capillaries compared to the control group, potentialized by physical exercise [49] even when EC was interrupted for 15 days [47]. However, Lee et al. [51] verified a significant decrease of angiogenic stimulator VEGF in the epicatechin group, followed by a slight reduction, although not significant, in the perimeter of the fiber compared with the control; however, the antiangiogenic factor TPS-1 did not increase in the EC group.

In the studies in humans that evaluated muscle function, positive effects were evidenced on the walk performance at a dosage of 75 mg of EC over six months [41] and on the increase of muscle strength at a dosage of 25 mg of EC for one week [48]. Mafi et al. [39] also obtained statistically significant results when epicatechin supplementation was combined with physical training. However, Schwarz et al. [38] observed aerobic adaptations with 400 mg/day of epicatechin supplementation but did not affect anaerobic training adaptations.

In humans, the acute ingestion of EC at 830 mg and 1245 mg dosages did not show any benefit to muscle recovery 24, 48, and 72 h after the exercise session [40]. Several studies have noted that the acute administration of cocoa polyphenols does not improve performance or post-exercise recovery [78,79,80,81].

The research in animals showed a difference in epicatechin dosages and administration time. In the functional analysis, walking performance was enhanced at the dosage of 1 mg/kg of EC for 8 weeks [49] and in physical activity using a dose of 0.25% for 37 weeks [52]. However, Ramirez-Sanchez et al. [55] observed a partial recovery of muscle strength when 1 mg/kg/day of EC was used for 30 days. In addition, Si et al. [44] obtained higher levels of AMPkα phosphorylation, suggesting that 0.25% of EC every other day for 15 weeks improves skeletal muscle function. Si et al. [52] also observed that epicatechin was able to delay muscle degeneration and improve physical activity at the dosage of 0.25% for 37 weeks. Additionally, Hüttemann et al. [45] observed an activation of AMPkα2 and an increase of fiber area in epicatechin groups.

Munguia et al. [54] used a higher dosage (2 mg of EC/kg) and obtained better results than the control in the functional test conducted in mice. Epicatechin has shown the capacity to increase the resistance to fatigue [33].

The literature has reported that higher dosages of EC in animals (4 mg kg/day for 24 days) inhibit the skeletal muscle adaptations at rest or during exercise as a result of blood flow impairment [82]. Nevertheless, Mi et al. [57] used a dose at 500 and 1000 mg/kg of EC in juvenile yellow river carp and noted a great enhancement of myogenic differentiation markers.

Oral dosages of 1–2 mg/kg of epicatechin do not cause adverse effects in animals [83]. However, dosages that represent more than 5% of the daily diet and are consumed for more than 3 months can produce acute cytotoxicity in liver cells, oxidative damage to pancreas DNA [84,85,86], and an enlargement of the thyroid [84].

Concerning the EC protocols, an important variability was identified in the studies with humans between 75 mg and 1245 mg [37,38,39,40,41,42,43]. In animals, the most frequently used dosage corresponded to 1.0 mg/kg, although the experimental time was divergent among the studies [44,45,46,47,48,49,50,51,52,53,54,55,56,57,58].

It is crucial to consider the risk of bias in each study included in this systematic review. First, smaller samples enhance the possibility of assuming a false premise as true. Reduced sample size was observed in Taub et al. [37], McDonald et al. [42], and Qureshi et al. [43]. A reduced evaluation time (5 days) was identified in Corr et al. [40], and the results did not show significant statistical differences. Reduced experimental time may lead to poor data quality or results restrictions. Furthermore, McDermott et al. [41] and McDonald et al. [42] did not carry out dietary assessment, an essential analysis that offers important results and improves the accuracy of intake. Regarding the experimental time, only Qureshi et al. [43], Hüttemann et al. [47], Si et al. [52], Gonzalez-Ruiz et al. [53], and Ramírez-Ramírez et al. [56] analyzed different time periods. Trial duration design of epicatechin supplementation is necessary, especially due to the low bioavailability of this polyphenol. Only Corr et al. [40], Mi et al. [57], and Moreno-Ulloa et al. [59] analyzed more than one dosage, providing dose-response information and more reliable results. Concerning the plasma concentrations of epicatechin, Taub et al. [37], McDermott et al. [41], and Ramirez-Sanchez et al. [46] performed this analysis. Drug concentration in plasma provides information about the half-life of epicatechin and the plasma concentration curve.

Another factor identified as a risk factor was the different periods of euthanasia reported by Si et al. [52]. Comparing the results of animals with variability in the period of euthanasia may not provide reliable data. Regarding the animal model, Si et al. [44] investigated the effects of epicatechin in db/db mice. The db/db mice have a shorter lifespan; therefore, other lineages should be considered in longevity studies. In addition, epicatechin interruption was evaluated only by Hüttemann et al. [47]. The washout period aims to evaluate a substance or drug’s residual effect (carry-over). Moreover, Gutierrez-Salmean et al. [48] did not report the participants’ gender, which represents a limitation since gender may influence the pharmacokinetics and effectiveness of drug treatment due to hormonal actions. Lastly, no control group was identified in one article Qureshi et al. [43]. Including a control group may provide a baseline in the experiment and reliable outcomes of the analyzed parameters because it validates the results of the study.

The limitations of this systematic review may be related to the search methodology used and to the restriction of the eligibility criteria. As a comprehensive view, this qualitative analysis presented a convergence of the positive effects of epicatechin on muscle growth and differentiation modulators.

Our systematic review highlights the promising potential of epicatechin in the context of muscular atrophy and its positive impacts in several biological models and systems. We recognize the need to identify gaps and guide future research directions.

When exploring the diversity of muscular atrophy models, the importance of a comprehensive characterization is crucial, considering molecular and functional nuances specific to each model. We commit to investigate additional models in order to capture a more complete picture of the effects of EC, including interactions between muscle fiber types and associated inflammatory responses.

In terms of the mechanisms involved, our current results emphasize the influence of EC in regulating protein synthesis and inhibiting muscle degradation. We agree that further investigation is crucial, with future studies focusing on elucidating specific signaling pathways, considering epigenetic aspects and intracellular inflammatory modulation.

Addressing the diversity of EC sources, is a priority that future research includes a more detailed analysis of variations in chemical compositions, aiming to better understand the specific bioactive profiles associated with each source.

We recognize the importance of randomized controlled clinical trials, committing to conducting a more rigorous review of the literature to identify and include clinical studies that meet these criteria. This will provide a more comprehensive and clinically relevant view of the effects of EC on muscle atrophy in humans.

In summary, our review, while offering valuable insights, is a starting point for future research. By addressing the questions raised, we plan to significantly contribute to the advancement of knowledge on the therapeutic application of EC in muscular atrophy.

Based on the above findings, future research prospects comprise the pressing need to establish standardized and well-defined therapeutic protocols for the administration of epicatechin in the treatment of muscle atrophy. Furthermore, conducting rigorous clinical studies with controlled and randomized designs is imperative to achieve more accurate therapeutic efficacy of epicatechin in humans. A comprehensive approach involving combined interventions, such as co-administration of epicatechin along with other therapeutic modalities, is required for further investigations. Thus, a deeper comprehension of the molecular mechanisms underlying the beneficial effects of epicatechin on muscle atrophy is essential to solidly establish clinical guidelines due to its promising application as a phytochemical “exercise pill”.

Finally, it is crucial to emphasize that more evidence is needed to expand the findings of this systematic review in order to provide concrete scientific results for the therapeutic use of epicatechin.

## 5. Conclusions

This systematic review provided important evidence concerning the effects of epicatechin on the regulation of the atrogenes (FOXO, MAFbx, and MuRF1) expression and the activation of the main myogenic regulator’s factors. The results evidenced the AKT/mTOR pathway signaling and mitochondrial biosynthesis induction, stimulated by epicatechin. Despite the discrepancies in the different parameters shown, the results are of great relevance due to the potential biological activities of such polyphenols. However, the scarce existing clinical studies are a barrier to validating EC’S therapeutic applicability in muscular atrophy-associated diseases.

## 6. Future Directions

Despite the biological properties of catechins, there are certain limitations for their clinical application, such as low bioavailability and degradation according to pH and temperature [87,88]. Nanotechnology could contribute by promoting the stability of catechins and prolonged release [89,90].

Nanodeliveries are biocompatible systems with physicochemical properties that increase bioavailability, pharmacokinetics, and pharmacodynamics. Some nanosystems used to encapsulate catechins include polymer nanoparticles, liposomes, lipids, proteins/peptides, gold nanocarriers, and liquid crystal nanocomposites [91,92].

The main challenge of nanotechnology is to develop delivery systems based on nanocarriers that target specific cells or tissues [93]. Also, it is necessary to consider the advantages and drawbacks of each nanoparticle system to guarantee the effectiveness of the therapeutic actions [94] and reduce the toxic effects of polyphenol overdose, allowing greater safety in its clinical application [92].

Furthermore, it would be interesting to study the behavior of epicatechin combined with other flavonoids, such as flavocoxid, a mixture of flavonoid containing baicalin and catechin, reported in the literature as an anti-inflammatory and antioxidant bioflavonoid, in addition to inhibiting muscle necrosis and improving the regeneration and function of skeletal muscles [95,96].

The authors emphasize the need to consider factors such as EC sources, doses, bioavailability, muscle atrophy models, supplementation period, and molecular/cellular mechanisms in future studies on the use of EC in the treatment of muscular atrophy.

A detailed analysis of chemical variations between EC sources is proposed, with an emphasis on bioactive profiles. Regarding dosage, a refined approach is suggested, considering bioavailability, with the proposal of a range of effective doses. For bioavailability, the importance of exploring formulations that optimize EC absorption in target tissues stands out, including studies on pharmacokinetics, such as lipid formulations.

Concerning muscle atrophy models, a comprehensive characterization is suggested, encouraging the exploration of additional models. Regarding the duration of supplementation, long-term studies are proposed to elucidate the temporal effects of EC, addressing genetic regulation and physiological adaptations. Expanding the discussion on molecular and cellular mechanisms, including epigenetic regulation and modulation of intracellular pathways, is considered crucial.

Finally, when integrating with physical training, it is suggested to investigate the synergistic interaction between EC supplementation and different exercise modalities, exploring combined effects. Finally, the urgency of methodological standardization stands out, proposing specific guidelines for collection, analysis, and presentation of results, involving uniformity and facilitating comparisons between studies.

## Figures and Tables

**Table 1:** Summary of the main EC supplementation parameters–Studies in humans.

First Author and Year	Manufacturer	Participants	Gender/ Age	Objective	Groups	Dosage	Experimental Time	Route of Administration	Procedure	Effects of EC (Main Results)
Taub et al., 2013 [37]	Hershey’s^®^ 60% Dark chocolate	5 participants	Male/ 47–71 years old	To evaluate the skeletal muscle growth with cocoa enriched with EC in patients with heart failure and type 2 diabetes.	-Control group: Patients aged 50–53 years with no disease. -Experimental group: Patients aged 47–71 years.	100 mg a day	3 months	Oral route	The patients underwent femoral quadriceps muscle biopsies before and after consuming cocoa enriched with EC.	There was a decrease in myostatin; however, it remained elevated compared to the control group. Follistatin increased above the controls with the treatment. The myogenin, MyoD, MEF2, and Myf5 levels were significantly stimulated with the EC treatment. *p* < 0.05
Schwarz et al., 2018 [38]		20 participants	Active men and women/Between 18 and 30 years old	To determine if EC supplementation increases the performance of cycling exercise.	EPI group or PLA (Placebo) group.	200 mg twice daily	4 weeks anaerobic and aerobic cycle training protocol.	Oral capsules	The cycling exercise sessions were conducted per week for 4 weeks (total of 16 sessions) with EC supplementation and a placebo.	EC supplementation did not affect myostatin expression but suppressed mitochondrial adaptations to exercise training. *p* ≤ 0.05
Mafi et al., 2019 [39]	Sigma-Aldrich, St. Louis, MO, USA	62 participants	Male /68 ± 2.86 years old	To evaluate the plasma levels of follistatin and myostatin in men with sarcopenia under training and EC supplementation.	RT: Resistance training, EP: EC, RT + EP: Resistance training + EC, PL: Double-blind placebo.	1 mg·kg^−1^·a day	8 weeks	Oral route(Daily capsules with 200 mL of water).	The training groups’ subjects conducted the protocol at 05:00 p.m. (45 min, 3 sets, 8–12 repetitions). The placebo group received starch capsules.	Follistatin significantly increased in the RT + EP groups compared to PL group. In comparison, myostatin reduced in the RT + EP and in RT groups. The maximum supine strength significantly improved in RT + EP and RT participants. *p* ≤ 0.05
Corr et al., 2020 [40]	Chococru^®^/EC	23 participants	13 women and 10 men/24 years.	To investigate if an acute dose of flavonoid cocoa (FC) may help in muscle recovery following EIMD.	CON: Control group: Did not receive FC, *n* = 8; CF830: High FC dose 830 mg group, *n* = 8; CF1245: FC overdose group 1245 mg, *n* = 7.	830 mg and 1245 mg	5 days (2 adaptation days and 3 days of EC)	Oral route	The EIMD protocol consisted in the hip fastening to the dynamometer at 85° of bending using straps to isolate the knee (5 series of 10 maximum concentric and eccentric contractions of the knee.	No significant modifications were observed between the groups for all the measures in the bending exercises. The FC did not show benefits in muscle recovery after 24 h, 48 h, and 72 h after EIMD protocol. *p* ≤ 0.05
McDermott et al., 2020 [41]	Hershey’s Co^®^.	44 participants	Male and female/≥60 years old.	To evaluate if cocoa with EC improves walking performance in aged people with peripheral artery disease.	Cocoa drink/Epi (*n* = 23) versus placebo drink (*n* = 21) (did not contain cocoa or EC).	75 mg	6 months	Oral route	The physical activity was conducted over 7 days with Accelerometer ActiGraph placed on the right hip.	Statistical differences were observed in the Cocoa/Epi group versus the placebo group in the 6-min walk test 2.5 h after consuming the drink. These results suggest a therapeutic effect of cocoa/Epi in the walk performance. However, cocoa/Epi did not significantly affect myostatin, follistatin, and Pax7. *p* < 0.10
McDonald et al., 2021 [42]	cGMP facility (Syngene, Karnatak, India)	7 participants	Male/18–60 years old	To evaluate EC capacity in mitochondrial biogenesis and in the muscle markers.	Nonrandomized clinical trial (before and after).	50 mg twice a day	8 weeks	Oral route (gelatin capsules).	The participants received two capsules in the morning and two in the evening. The brachial biceps muscle biopsies were collected pre- and post-treatment.	Follistatin significantly increased, while myostatin decreased. There was a significant increment of tissue markers Myf5, MyoD, myogenin, and MEF2a. EC stimulated PGC1α (a coactivator of mitochondrial biogenesis). *p* < 0.05
Qureshi et al., 2021 [43]	Epirium Bio, Inc.	10 participants	Both/10 to 22 years old	To analyze the efficacy of EC in patients with Friedreich’s ataxia.	Prospective, nonrandomized, open-label study	75 mg/daily	12 and 24 weeks	Oral route	Subjects received 25-mg capsule, 3/daily (75 mg daily) to assess clinical and biochemical parameters. Mitochondrial function pre- and post- EPI treatment and oxidative damage were measured.	Follistatin was higher at 12 and 24 weeks after consumption (12 weeks, *p* = 0.020; 24 weeks, *p* = 0.016). However, myostatin levels demonstrated no significant differences at 12 or 24 weeks. *p* < 0.05

Abbreviations: Myogenic factor 5 (Myf5); Myoblast determination protein 1 (MyoD); Myocyte Enhancer Factor 2A (MEF2a). Exercise-induced muscle damage (EIMD); Flavonoid cocoa (FC); Paired box protein (Pax-7). Myogenic factor 5 (Myf5); Myoblast determination protein 1 (MyoD); Myocyte Enhancer Factor 2A (MEF2a); Peroxisome proliferator-activated receptor coactivator-1 (PGC-1).

**Table 2:** Summary of the main EC supplementation parameters–Studies in animals.

First Author and Year	Manufacturer	Population	Gender/Age	Objective	Groups	Dosage	Experimental Time	Route of Administration	Procedure	Effects of EC (Main Results)
Si et al., 2011 [44]	Sigma-Aldrich	29 C57BLKS/J and KS.Cg-m +/+Lepr db/J, db/db Mice	Male/ 5 weeks of age	To investigate the effects of EC in obese diabetic mice.	Con: *n* = 12 Control group: C57BLKS/J Mice; db: *n* = 6: Diabetic rats without EC. db + EC: *n* = 11: 0.25%: Diabetic rats + EC.	0.25% every other day	15 weeks	Oral route	To determine the contractile function, the EDL muscles were excised and attached by means of a suture to a servomotor (Aurora Scientific).	EC significantly decreased the inflammatory markers (C-reactive protein) in diabetic rats. The GSK antioxidant concentration and AMPKa phosphorylation were considerably higher than db group. *p* < 0.05
Hüttemann et al., 2012 [45]	Sigma Aldrich, USA	C57BL/6, *n* = 32	Male mice/5-month-old	To determine whether EC could enhance endurance capacity on detraining hindlimb muscles of mice.	Four groups: Group 1: Control group; Group 2: Trained rats; Group 3: mice were trained + a detraining period of 14 days. Vehicle (water) (DT-14-W); Group 4: Trained + 14 days of detraining. EC treatment (DT-14-Epi).	1 mg/kg twice daily	14 days	Oral gavage	Groups 2, 3, and 4 performed a training (treadmill) 5 times a week for 5 weeks with a pre- and post-training analysis 48 h after the exercise test. Animals underwent a third incremental treadmill test. The plantaris and quadriceps femoris muscles were collected for analysis.	In the DT-14-W and DT-14-Epi (groups 2 and 3), the VEGF-A protein was higher compared to groups 1 and 2. Complex I expression was increased in the DT-14-Epi group compared to the group 1. However, the expression of complex III protein was significantly greater in the group 4. The fiber area was greater in the trained and group 4. *p* ≤ 0.05
Ramirez-Sanchez et al., 2012 [46]	Sigma-Aldrich	25 C57BL/6N	Male mice/One-year-old	To examine the Epi effect on cardiac angiogenesis and plantaris muscle when Epi and exercise are combined.	Four groups: (1) Water; (2) Water exercise (W-Ex); (3) EC (Epicatechin) and (4) EC exercise (Epi Ex).	1 mg/kg twice a day	15 consecutive days	Oral gavage	All animals exercised on a treadmill at a slow speed and at 10° inclination angle for 5–10 min until exhaustion. Plantaris muscle was collected for further analysis.	Plantaris muscle capillary was increased by EC. VEGF protein was significantly enhanced by Epi and Exercise alone, but when combined, VEGF was enhanced (10%). p-PI3K was increased further on the Epi-Ex group (~80%). *p* ≤ 0.05
Hüttemann et al., 2013 [47]	Sigma- Aldrich	21 LCR rats (rats grown for low capacity to run) with congenital muscle dysfunction.	Males/5 months of age	To determine the action of EC on angiogenesis and mitochondrial proliferation.	Control: Water group for 30 days; Epi 30d: EC for 30 days; post-Epi 15d: EC for 30 days and 15 days without EC.	1 mg/kg twice a day	EC for 30 days, followed by 15 days without EC.	Gavage	The plantar muscle was analyzed in order to determine the effects of EC on a glycolytic muscle fiber.	EC increased in capillarity and mitochondrial biogenesis in the 15-day treatment period, including in the 15-day period of treatment interruption. EC increased VEGF and reduced CD47 and the receptor TSP1, and it also activated the P38 MAPK pathways. *p* ≤ 0.05
Gutierrez-Salmean et al., 2014 [48]	Sigma-Aldrich	20 C57BL/6 Mice *n* = 20 5/group	Young males/6 months and senile males/26 months	To examine the changes to the protein levels in the skeletal muscle of young vs senile humans and mice.	Ctrl (Young), Epi (Senile), Ctrl (Senile), Epi (Young)	1 mg/kg	2 weeks	Gavage	The control groups received water through gavage. Quadriceps muscle samples were obtained from the mice.	Epicatechin significantly decreased the myostatin levels 15% (young) and 21% (aged). Follistatin increased 56% in the senile group. Myogenin significantly increased in young and senile animals (16%, 21%, respectively), while MyoD increased 19% in senile rats. Myf5 incremented 12% (young) and 15% (senile), and MEF2 10%, 19%, respectively. *p* < 0.05
		12 participants	Gender not reported/Young adults: 28 years old, *n* = 6 Aged: 62 years old, *n* = 6	To evaluate the effects of the treatment with epicatechin on muscle strength and on the plasma levels of myostatin and follistatin.	Young adults’ group (*n* = 6) Senile group (*n* = 6)	25 mg/day	1 week	Oral route (capsule)	The muscle strength was evaluated by hand grip dynamometry (three times with each hand, alternating the hand and resting for 10 s to prevent fatigue).	The treatment with epicatechin increases the hand’s muscle strength by 7%. With age, there was a significant increase in myostatin (28%, 48%). The treatment with EC significantly increased the plasma levels of follistatin (49%). *p* < 0.05
Lee et al., 2015 [49]	Sigma-Aldrich, St. Louis, MO, USA	34 C57BL/6N Mice	Males/14 months of age	To determine the effect of epicatechin on angiogenesis and mitochondrial biogenesis protein markers.	C: control group; CE: control with resistance training; Epi: epicatechin; Epi-Ex: epicatechin + training.	1 mg/kg twice a day	8 weeks	Gavage	The training groups’ mice were submitted to training on a treadmill for 8 weeks (5 times/week for 60 min/session).	The Epi-Ex showed better resistance performance, and a significantly higher VEGF-R2 expression, and increased PGC-1b and TFAM. FoxO1 expression was reduced in the experimental groups. *p* ≤ 0.05
Moreno-Ulloa et al., 2015 [50]	Not reported	15 C57BL/6	Male mice/26-month-old	To compare the protein levels in senile mice versus young mice on skeletal muscle, heart, kidney, and brain.	(1) Y mice: (6-month-old), *n* = 5; (2) S mice: (26-month-old), *n* = 5; (3) S mice: treated with EC, *n* = 5.	1 mg/kg twice daily	2 weeks	Gavage	Muscle biopsy tissue was processed for analysis.	EC re-establish GSH in skeletal muscle (SkM). Aging biomarkers were reduced in old mice. In SkM, Epi administration increased complex I protein levels (C-I) and significantly decreased SA-β-gal protein. *p* < 0.05
Lee et al., 2016 [51]	Sigma-Aldrich, St. Louis, MO, USA	25 C57BL/6N Mice	Males/6 months of age	To determine if the treatment with EC may mitigate the muscle mass loss in skeletal muscle.	C: Control (water); HS-V: Suspension of the hind limbs + water; HS-EC: Suspension of the hind limbs + EC.	1.0 mg/kg twice a day (Morning and evening).	14 consecutive days	Gavage	For the hind limb suspension protocol, the animals were placed in a cage with a steel bar. The soleus, medial, and gastrocnemius muscles were removed from both hind limbs.	HS-EC showed significantly higher FCSA. In HS-Epi there was a slight decrease in FP compared to the control group. VEGF-A was lower in the vehicle or epicatechin groups. HS-Epi showed a significant increase in mTOR, Akt, and TFAM. PGC-1β was only induced in HS-Epi, and CcO was similar to the control. FoxO and GSK-3β were induced in HS-V. *p* ≤ 0.05
Si et al., 2019 [52]	Millipore Sigma, Burlington, MA, USA	33 C57BL/6 Mice	Males/ 9 months and 20 months of age	To investigate the effects of EC on the survival rate and on the physical performance in aged mice.	OC: Control (aged mice); YC: Young control: 9-month-old mice; EC: 0.25% epicatechin.	0.25%	37 weeks and 44 weeks	Oral route	The samples were collected following 37 weeks, and the rest was treated for one additional week (on week 44).	EC attenuated the deterioration of the muscle; in addition, it improved physical activity, and delayed the degeneration of the quadriceps. E in senile mice presented a survival rate (69%) compared to the control group (39%). *p* < 0.05
Gonzalez-Ruiz et al., 2020 [53]	Sigma-Aldrich	36 Long-Evans Rats	Females/11 weeks	To analyze the effects of epicatechin on the regulation of UPS proteins in the hind limbs.	SCI + water 7 days: *n* = 6; SCI + Epi 7 days: *n* = 6; SCI + water 30 days: *n* = 9; SCI + Epi 30 days: *n* = 9; Sham: Only laminectomy *n* = 6.	1 mg/kg/day	1 week and at 30 days	Gavage	The spinal cord was sectioned (region of the T8 to T10 vertebrae). The left side gastrocnemius and soleus muscles were dissected.	At 30 days, the injury group lost 49.52% of the cross-sectional area of the muscles, and the epicatechin groups lost 24.28 ± 15.45%. After 7 days, the SCI + EC had only one significant difference in MuRF. The treatment with EC induced a significant decrease in atrophy markers FOXO, MAFbx, and MuRF1 compared to the control group (VEH) after 7 and 30 days from the lesion. *p* < 0.05
Munguia et al., 2020 [54]	Sigma-Aldrich Co. (St. Louis, MO, USA)	15 C57BL/6 Mice induced to a high-fat diet	Males/10 weeks	To evaluate the benefits of the flavonoids in the improvement of the physical activity decreased by age/high-fat diet.	Three interventions: Control: Water; High-flavonoid dark chocolate; (DC) drink: 2 mg EC + 12.8 mg procyanidins/kg); EC: Epicatechin (2 mg EC/kg).	2 mg EC/kg	5 weeks of treatment with EC. Week 64–Change from normal diet + 5 weeks of treatment. Total: 69 weeks.	Gavage	Gastrocnemius were collected. The inverted screen and front limbs functional test consisted in the longest time hanging, establishing a fixed time of 120 s and 130 s, respectively.	EC increased follistatin and myocyte enhancer factor 2A (MEF2A) expression. DC and EC decreased FoxO and MURF; however, MAFbx was not significant. DC and EC reduced the fat content and increased physical performance compared to the control. *p* < 0.05
Ramirez-Sanchez et al., 2021 [55]	Sigma-Aldrich, Inc./Hershey, PA, USA	30 Wistar Rats	Male/3 months of age	To examine the potential restorative effects of epicatechin in muscular atrophy-induced rats.	Control group (*n* = 15): Without physical restriction (water): The experimental group (*n* = 15): Physical restriction (2 weeks). Rats were divided into two groups: Epi GWI-Epi group (*n* = 8) and Water GWI group (*n* = 7).	1 mg/kg/day	2 weeks of EC. Atrophy induction (3 weeks) + 1 maintenance week + 2 weeks of EC. On week 6–Functional test and euthanasia.	Gavage	Atrophy induction protocol: pyridostigmine bromide (PB) 1.3 mg/kg/day through the oral route, permethrin 0.13 mg/kg/day, and DEET 40 mg/kg/day. The animals were physically contained for 5 min/day for 3 weeks.	The treatment with epicatechin induced a partial recovery of muscle strength and run distance on treadmill. MURF, Fbox40, and atrogin-1 were partially recovered by EC. Epicatechin significantly increased AKT and mTORC1 activation. *p* < 0.05
Ramírez-Ramírez et al., 2022 [56]	Sigma-Aldrich, St. Louis, MO, USA	One hundred twenty-six 132 CD-1 mice	Male /10-weeks-old	To examine the effects of EC treatment in the Tibialis anterior muscle repair process.	Two treatments: Vehicle treatment: right leg injured with BaCl2 (WI-E) and left leg without damage (WOI-E). EC treatment: right leg injured with BaCl2 (WI + E) and left leg without injury (WOI + E).	1 mg/kg EC/kg	C was administrated every 12 h and animals were sacrificed at 12 and 24 h, 2 days, 4 days and 15 days.	Oral gavage twice daily	Hind legs tibialis anterior muscles were collected for histological analyses.	EC significant increased MyoD and Myogenin at 24 h (h) after injury compared to the other groups. Histological lesion in WI + E presented a smaller lesion area after 24 h (*p*= < 0.05), and also more significant reduction after two days (*p* = 0.0149). The number of central nuclei were increased only at 12 h post-injury in WI + E. *p* < 0.05
Mi et al., 2023 [57]	Nanjing Daosifu Biotech Co., Ltd., Nanjing, China.	300 fish (16.27 ± 0.24 g).	Not reported/Juvenile yellow river carp.	To investigate the antioxidant and muscle fiber growth effects of EC.	The groups were divided according to the amount of epicatechin present in the diet, as follows: EC (0, 100, 500, and 1000 mg/kg).	0, 100, 500, and 1000 mg/kg.	The juvenile carp were fed three times a day for 60 days	Hand-fed	Juvenile carp were randomly allocated in 3 tanks per group. Four blocks of muscle were collected from the bilateral dorsal fin.	EC activated AMPKα2 and PGC-1α. EC 500 and EC 1000 groups increased muscle hardness and SOD activity. EC 1000 group upregulated MyoD, and myogenin and downregulated Myostatin b (mstnb). *p* < 0.05
Palma-Flores et al., 2023 [58]	Sigma-Aldrich, St. Louis, MO, USA	Twelve CD-1 mice	Not reported/2.5 months old	To determine the potential activity of epicatechin on the expression of miRNAs in skeletal muscle growth and regeneration.	Two groups: Control, Ctrl: Water-treated and Epi-treated (Epi).	1 mg/kg EC/kg	Two weeks	Oral gavage twice daily	After treatment, the quadriceps muscles samples were excised and stored for further analysis.	MyoD and myogenin were increased by EC. *p* < 0.05

Abbreviations: Extensor digitorum longus muscle (EDL); Glutathione (GSH); AMP-activated protein kinase (AMPKα); Vascular endothelial growth factor (VEGF); Mitochondrial Respiratory Complex I and III. EC (EC/Epi); Endothelial growth factor (VEGF); Phosphoinositide 3-kinase (PI3K); Differentiation Cluster 47 (CD47); Thrombospondin-1 (TSP-1); Mitogen activated protein kinase (MAPK). Myogenic factor 5 (Myf5); Myoblast determination protein 1 (MyoD); Myocyte enhancer factor 2A (MEF2A). Vascular endothelial growth factor (VEGF); Peroxisome proliferator activated receptor coactivator 1 (PGC-1); Forkhead transcription factors family (FoxO); Mitochondrial transcription factor A (TFAM); Epicatechin (EC/Epi); Glutathione (GSH); Skeletal muscle (SkM); Mitochondrial Respiratory Complex I and III (C-I and C-III); Senescence-associated beta-galactosidase (SA-beta-gal). Vascular endothelial growth factor receptor 2 (VEGF-R2); Fiber cross-sectional area (FCSA); Fiber perimeter (FP); Forkhead transcription factors family (FoxO); Thrombosponding antiangiogenic factor (TPS-1); Mitochondrial transcription factor A (TFAM); Protein kinase B (AKT); Mammalian target protein of rapamycin (mTOR); Peroxisome proliferator-activated receptor coactivator-1 (PGC-1); Cytochrome c oxidase (CcO); Enzyme Glycogen Synthase Kinase 3 Beta (GSK-3b). Ubiquitin proteasome system (UPS); Dark chocolate drink (DC); Forkhead transcription factors family (FoxO); F-box muscular atrophy (MAFbx); Muscle RING-finger protein (MuRF1); Myocyte enhancer factor 2A (MEF2A); Epicatechin (EC). Epicatechin (Epi/EC); Gulf War Illness (GWI); Pyridostigmine bromide (PB); N, N-dimethyl-meta-toluamide (DEET); Muscle RING-finger protein (MuRF1); F-box muscular atrophy (MAFbx); Protein kinase B (AKT); Mammalian target protein of rapamycin (mTOR); Salts of barium (BaCl2); Myoblast determination protein 1 (MyoD). Epicatechin (Epi/EC); AMP-activated protein kinase (AMPKα); Peroxisome proliferator-activated receptor coactivator-1 (PGC-1); Superoxide dismutase (SOD); Myoblast determination protein (MyoD).

**Table 3:** Summary of the main EC supplementation parameters–Studies in culture cells.

First Author and Year	Manufacturer	Type of Muscle Cells	Objective	Groups	Dosage	Experimental Time	Procedure	Effects of EC (Main Results)
Moreno-Ulloa et al., 2018 [59]	EPI, MISSION^®^ siRNA Universal Negative Control #1	C2C12 myoblasts	To analyze if EC stimulates mitochondrial biogenesis (MiB).	Control group: Dimethyl sulfoxide DMSO used as vehicle; Epi 3 µm: Treatment with 3 µm of EC; Epi 10 µm: Treatment with 10 µm of epicatechin.	EPI (3 µM and 10 µM	48 h	Myotubes in DMEM were treated with an incubation time of 48 h.	COX-I/SDH-A was increased by epicatechin, indicating the effect of Epi on mitochondrial biogenesis. Epi increased the width and length of C2C12 myotubes. *p* ≤ 0.05
Ortiz-Flores et al., 2020 [60]	Merck KGaA, Darmstadt, Germany	Mouse myoblast (C2C12 cells)	To demonstrate that EC probably activates PXR as a target in C2C12 myoblasts.	Control: FBS 10%; Positive control: 2% horse serum EC: 1 μM; EC + Keto: 1 μM + 10 μM ketoconazole (PXR’s antagonist); PCN (PXR activator): 1Μm of Pregnenolone-16a-carbonitrile (PCN); PCN + Keto: 1 μM of PCN + 10 μM ketoconazole.	EC 1 μM	30 min	C2C12 cells were cultured in DMEM-F12. After C2C12 differentiation assay, myogenin was quantified.	EC activated PXR, promoting muscle cell differentiation and increasing myogenin and Cyp3a11 expression in C2C12 cultured cells. *p* < 0.05
Edwards et al., 2022 [61]	Epi: #E1753 Sigma	Mouse skeletal muscle C2C12 myoblasts	To investigate the effects of EC and HA (hippuric acid) on skeletal muscle morphology and metabolism investigating an in vitro model of muscle atrophy with dexamethasone.	Divided into 6 groups: VC-CTRL: vehicle control. VC-DEX: cells incubated in dexamethasone; EPI-CTL: cells incubated with 25 μM EC; EPI + DEX: cells were incubated in 25 μM EC and 100 μM DEX; HA-CTL: cells incubated in 25 μM HA.; HA + DEX: cells incubated with 25 μM HA and 100 μM DEX	25 μM EPI and 100 μM DEX.	24h of treatment protocol	Cells were incubated in DMEM (5mM glucose), followed by 6 days of differentiation, and received 24 h of treatment.	PGC1 α, ACC, and TFAM (regulators of mitochondrial function) were significantly lower in DEX-treated versus CTL cells (Control). However, EPI or HA partially attenuated the proteolysis in DEX-treated groups by preserving the expression of LC3 and caspase-3 protein. Myotube diameter was significantly greater in EPI-DEX and HA- DEX. *p* ≤ 0.05

Abbreviations: Epicatechin (Epi/EC); Dulbecco’s Modified Eagle Medium (DMEM); Epicatechin (EPI/EC); Cytochrome C Oxidase/Succinate Dehydrogenase (COX-I/SDH-A); The pregnane X receptor (PXR); Pregnenolone-16a-carbonitrile (PCN); Cytochrome P450 family 3 subfamily A (CYP3A). Epicatechin (EPI/EC); Dexamethasone (DEX); Hippuric acid (HA); Dulbecco’s Modified Eagle Medium (DMEM); Peroxisome proliferator-activated receptor coactivator-1 (PGC-1); Acetyl-CoA carboxylase (ACC); Mitochondrial transcription factor A (TFAM); Light chain 3 (LC3).

**Table 4:** Risk of bias of the studies.

Risk of Bias	Taub et al., 2013 [37]	Schwarz et al., 2018 [38]	Mafi et al., 2019 [39]	Corr et al., 2020 [40]	McDermott et al., 2020 [41]	McDonald et al., 2021 [42]	Qureshi et al., 2021 [43]	Si et al., 2011 [44]	Hüttemann et al., 2012 [45]	Ramirez-Sanchez et al., 2012 [46]	Hüttemann et al., 2013 [47]	Gutierrez-Salmean et al., 2014 [48]	
Reduced sample size	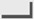					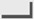	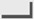						
Reduced evaluation time				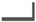									
Failure to collect the participants’ diet					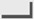	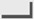							
Only one period was evaluated	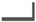	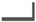	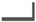	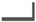	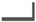	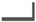		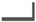	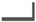	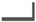		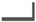	
Only one dose was studied	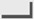	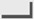	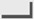		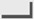	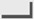	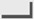	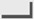	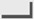	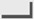	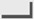	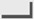	
Absence of plasma (epicatechin) measured.		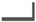	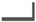	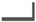		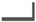	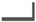	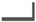	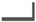		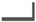	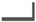	
Difference of the euthanasia periods													
Choice of the animal model								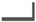					
Epicatechin interruption was not evaluated	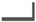	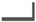	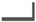	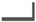	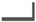	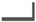	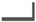	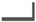	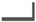	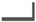		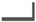	
Participants’ gender not reported												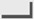	
No control group							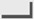						
Reduced sample size													
Reduced evaluation time													
Failure to collect the participants’ diet											N/A	N/A	N/A
Only one period was evaluated	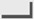	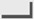	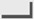			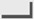	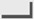		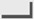	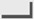	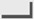	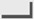	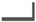
Only one dose was studied	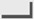	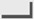	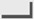	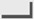	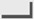	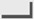	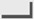	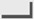		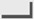		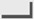	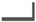
Absence of plasma (epicatechin) measured.	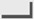	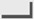	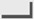	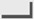	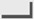	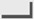	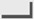	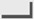	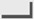	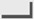	N/A	N/A	N/A
Difference of the euthanasia periods				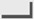							N/A	N/A	N/A
Choice of the animal model											N/A	N/A	N/A
Epicatechin interruption was not evaluated	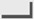	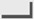	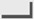	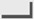	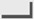	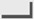	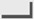	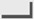	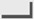	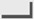			
Participants’ gender not reported											N/A	N/A	N/A
No control group													
Risk of Bias	Lee et al., 2015 [49]	Moreno-Ulloa et al., 2015 [50]	Lee et al., 2016 [51]	Si et al., 2019 [52]	Gonzalez-Ruiz et al., 2020 [53]	Munguia et al., 2020 [54]	Ramirez-Sanchez et al., 2021 [55]	Ramirez- Ramírez et al., 2022 [56]	Mi et al., 2023 [57]	Palma-Flores et al., 2023 [58]	Moreno-Ulloa et al., 2018 [59]	Ortiz-Flores et al., 2020 [60]	Edwards et al., 2022 [61]
Reduced sample size													
Reduced evaluation time													
Failure to collect the participants’ diet											N/A	N/A	N/A
Only one period was evaluated	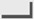	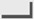	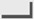			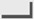	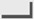		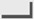	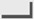	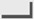	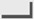	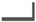
Only one dose was studied	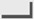	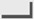	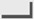	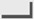	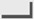	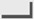	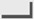	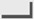		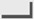		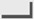	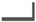
Absence of plasma (epicatechin) measured.	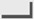	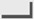	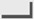	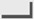	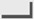	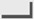	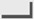	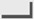	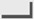	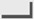	N/A	N/A	N/A
Difference of the euthanasia periods				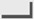							N/A	N/A	N/A
Choice of the animal model											N/A	N/A	N/A
Epicatechin interruption was not evaluated	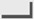	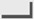	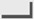	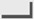	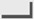	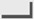	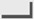	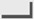	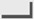	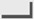			
Participants’ gender not reported											N/A	N/A	N/A
No control group													

Abbreviations: not applicable (N/A)—Cell culture studies. Symbol in the table presents the risk of bias.

## Data Availability

Data are contained within the article.
